# Spiritual Well-Being as a Patient-Reported Assessment Dimension in Advanced Cancer: Clinical Validation and Quality-of-Life Implications

**DOI:** 10.3390/healthcare14162525

**Published:** 2026-08-13

**Authors:** Diana-Maria Puscasu, Ioana Creanga-Murariu, Eliza-Maria Armeanu, Diana Monor, Ioan Matei Rusu, Bogdan Gafton, Vladimir Poroch, Teodora Alexa-Stratulat

**Affiliations:** 1 Department of Medical Genetics, Faculty of Medicine, Grigore T. Popa University of Medicine and Pharmacy, 700115 Iasi, Romania; 2 Advanced Research and Development Center for Experimental Medicine (CEMEX), Grigore T. Popa University of Medicine and Pharmacy, 700115 Iasi, Romania; 3 Oncology-Radiotherapy Department, Grigore T. Popa University of Medicine and Pharmacy, 700115 Iasi, Romaniateodora.alexa-stratulat@umfiasi.ro (T.A.-S.); 4 Centre for Translational Medicine, Semmelweis University, 1085 Budapest, Hungary; 5 Department of Medical Oncology, Regional Institute of Oncology, 700483 Iasi, Romania; 6 2nd Internal Medicine Department, Faculty of Medicine, Grigore T. Popa University of Medicine and Pharmacy of Iasi, 700115 Iași, Romania; 7 Department of Psychology, Faculty of Psychology, Alexandru Ioan Cuza University, 7000756 Iasi, Romania; 8 Department of Palliative Care, Regional Institute of Oncology, 700483 Iasi, Romania

**Keywords:** spiritual well-being, patient-reported assessment, EORTC QLQ-SWB32, clinical validation, palliative care, quality of life

## Abstract

**Background**: In advanced cancer care, routine clinical assessment often focuses on symptoms, treatment tolerance, and disease status, while spiritual well-being remains insufficiently explored despite its relevance to quality of life (QoL). Standardized patient-reported tools may help identify spiritual and existential vulnerability in patients receiving palliative treatment. This study evaluated the EORTC QLQ-SWB32 as a structured spiritual well-being assessment tool in advanced cancer care through its cultural adaptation and clinical validation among Romanian patients. Furthermore, it evaluated the tool’s feasibility and explored the association between spiritual well-being and QoL in individuals receiving palliative systemic therapy. **Methods:** Following EORTC Quality of Life Group guidelines, the QLQ-SWB32 was translated and pilot-tested with ten cancer patients. A prospective, cross-sectional observational study was then conducted at the Regional Institute of Oncology in Iași, Romania. Over six months, 42 adult patients with metastatic or locally advanced inoperable malignancies undergoing palliative systemic therapy were enrolled. Participants completed the EORTC QLQ-SWB32 and EORTC QLQ-C15-PAL questionnaires, while sociodemographic and clinical variables were collected using structured case report forms. **Results:** The translated instrument showed good linguistic clarity and clinical acceptability during pilot testing. The study cohort was predominantly aged 40–64 years (52.38%) and was socio-culturally homogeneous, with all patients identifying as Romanian and most as Eastern Orthodox (92.85%). The Relationship with God/the Divine (RSG) domain showed a stronger association with QoL in male patients than in female patients, particularly in subsequent treatment lines. In the subsequent-line subgroup, residential environment reached statistical significance (*p* = 0.021), with a stronger RSG–QoL correlation among rural residents. Age-related inversion patterns in the RSG–QoL association were also observed across treatment lines. **Conclusions:** Spiritual well-being assessment may provide clinically relevant patient-reported information in advanced cancer care. The Romanian EORTC QLQ-SWB32 demonstrated feasibility, linguistic clarity, and clinical validity as a structured assessment tool. The association between the Relationship with God/the Divine domain and QoL varied according to sex, age, residential environment, and treatment trajectory, supporting the integration of standardized spiritual assessment into individualized palliative oncology care.

## 1. Introduction

The increasing global burden of cancer represents a major threat to quality of life (QoL), affecting patients across physical, psycho-emotional, social, and spiritual domains [1]. In parallel with advances in oncological care, the conceptual framework of cancer management has progressively shifted from a disease-centered model focused primarily on physical outcomes toward a more comprehensive, patient-centered approach. This contemporary perspective recognizes that QoL is shaped not only by symptoms and treatment-related toxicity, but also by psychosocial and spiritual factors that influence how patients experience and adapt to illness [2].

As therapeutic progress has improved overall survival (OS) in many cancer settings, the number of patients living with advanced or incurable disease has increased substantially. European Commission data (2025) indicate that female cancer mortality declined by 7% between 2011 and 2021, this reduction was slightly lower than the European Union (EU) benchmark (9% decline), yet aligned with the average observed across Romania’s economic peer nations (7% decline). Similarly, while male cancer mortality in Romania declined by 8% in the same period, this reduction remained substantially lower than the EU-wide average (16%) and the average for economic peers (12%) [3]. Consequently, preserving and improving QoL has become a central objective in palliative oncology, alongside disease control and symptom management. This is particularly relevant in patients receiving palliative systemic therapy, whose life expectancy may be prolonged by modern anticancer treatments and supportive care strategies, but who remain exposed to cumulative physical, emotional, and existential distress [4].

Although the World Health Organization (WHO) conceptualized health as a “state of complete physical, mental, and social well-being” as early as 1947, the construct of QoL became widely integrated into medical literature only in the 1960s and subsequently emerged as an essential outcome in the management of patients with incurable diseases [5]. Over time, QoL has been interpreted through several complementary frameworks, ranging from the fundamental “joy of living” [6] to an individual’s subjective perception of their position in life, shaped by cultural context, personal expectations, and value systems [7]. In clinical oncology, QoL also encompasses psychological and social well-being, functional autonomy, the capacity to maintain daily activities, and the ability to adapt to disease-related stressors and treatment-induced adverse effects [8]. From a health-centered perspective, QoL may therefore be understood through two major dimensions: functional status in everyday life and subjective satisfaction derived from these activities [9].

Within this multidimensional framework, spirituality represents an important but frequently under-assessed component of QoL. The spiritual dimension explores the relationship between spiritual needs and physical, mental, and social health outcomes, and is considered a key element of the holistic biopsychosocial model. Although relevant across several terminal conditions, the spiritual dimension of QoL has been most extensively investigated in patients with cancer, in whom diagnosis, disease progression, and treatment burden often generate profound existential concerns [10].

Spirituality refers to an individual’s relationship with the self, others, nature, life, and a transcendent reality, which may be understood as God, Spirit, the transcendent Self, Nature, or the Universe. In patients with advanced malignancies or limited prognosis, spirituality may influence QoL by shaping coping strategies, emotional adaptation, the interpretation of suffering, and the search for meaning during illness. For this reason, spiritual well-being is increasingly regarded as an integral component of holistic cancer care rather than a peripheral or exclusively private concern [11,12].

According to the European Organisation for Research and Treatment of Cancer (EORTC) Quality of Life Group, spiritual well-being includes several dimensions: the relationship with self and others, the existential dimension, and the relationship with divinity as expressed through personal beliefs or religious practices [13]. Over the past two decades, research has increasingly examined the association between spirituality, clinical status, and QoL in patients with cancer. These investigations are grounded in the observation that patients with incurable malignancies frequently experience heightened spiritual needs, including the search for existential meaning, reconciliation with the self and others, and the need to preserve a sense of inner coherence despite disease progression [14]. Following diagnosis, many patients turn toward spiritual or religious frameworks as coping mechanisms during the disease trajectory and its treatment; notably, more than 80% of patients with advanced-stage illness report significant spiritual needs [15].

The WHO emphasizes that spiritual dimensions and religious practices should be addressed as part of medical care, particularly in chronic and incurable diseases. However, the clinical assessment of spiritual needs in patients with terminal illness remains challenging. These difficulties arise not only from the sensitive and intimate nature of spirituality, but also from the conceptual ambiguity of the construct. In clinical practice, it is often necessary to distinguish between formal religious affiliation, communal religious practices, and broader spiritual concerns that may also be present in patients who do not identify with a specific religious tradition [16].

Evidence suggests that, in newly diagnosed patients with advanced cancer, spirituality may be a stronger predictor of overall QoL than social well-being, emotional well-being, or physical status. This finding highlights the depth of spiritual and existential needs in this population and supports the need for a more systematic clinical approach to this dimension [17,18]. When spiritual concerns remain unrecognized or unresolved, patients may develop spiritual distress, a form of profound suffering that may require additional resources, specialized support, and targeted interventions [19].

Several instruments have been developed to assess spiritual well-being in clinical populations. Among them, the EORTC Spiritual Well-Being Questionnaire evaluates spiritual fulfillment across four domains: interpersonal relationships, the relationship with self, the relationship with Divinity, and existential perspectives [20]. Such instruments provide a structured method for translating a complex subjective experience into a patient-reported assessment that can be integrated into clinical care.

In the context of patient-centered oncology, spiritual well-being is increasingly recognized as a core dimension of comprehensive palliative management and is closely intertwined with QoL. Nevertheless, the systematic integration of spiritual well-being assessment into routine advanced cancer care remains limited. In Romania, this gap is further amplified by the limited clinical validation of culturally and linguistically adapted instruments for assessing spiritual well-being in patients with advanced malignancies. This context is particularly relevant in a population in which religious identity and spiritual coping may substantially influence the patient’s experience of illness.

The assessment of QoL is an essential component of cancer care, as the diagnosis and treatment of cancer exert long-term effects across multiple dimensions of human health [21]. However, clinical interactions between oncologists and patients often remain focused primarily on objective signs, symptoms, laboratory findings, imaging results, and treatment-related toxicity. As a consequence, broader dimensions of well-being may be insufficiently explored, and the improvement of general well-being may be perceived as secondary to disease-directed outcomes rather than as a central component of care [22].

This issue is particularly important in healthcare systems where palliative care and psycho-oncology services are unevenly developed. In Romania, palliative care infrastructure remains insufficient and geographically disproportionate, with suboptimal resources in several regions. In addition, psycho-oncology services are limited by a shortage of specialized professionals and by insufficient organizational integration into routine cancer care [23].

According to published data, a major barrier exists regarding access to screening programs, as evidenced by low screening rates for breast and colorectal cancers. This leads to diagnosis at more advanced stages, primarily driven by a shortage of specialized medical personnel and an uneven geographical distribution of healthcare facilities across the country [3]. Current analytical data also indicate that cancer mortality rates in Romania exceed the European Union average, while overall cancer management performance remains below European benchmarks [24]. Data from a 2019 national evaluation highlights critical infrastructural deficits in Romania’s palliative care delivery, meeting an estimated 5% of the overall demand. Hospitalization served as the predominant care modality for 29,650 out of 33,000 patients in need, reflecting systemic limitations in community-based alternatives. Home-based palliative care accounted for only 5% of service provision, constrained by the underdevelopment of public specialized infrastructure and its primary restriction to private healthcare entities [3]. These structural challenges reinforce the need for simple, standardized, and clinically applicable tools capable of identifying unmet supportive care needs in patients with advanced cancer.

The novelty of this study lies in the integration of spiritual well-being into the structured clinical assessment of patients with advanced cancer. Rather than approaching spirituality solely as a subjective or psychosocial construct, this study evaluates spiritual well-being as a patient-reported dimension that may help identify QoL vulnerability across different stages of the palliative treatment trajectory. By examining the association between spiritual well-being and QoL according to sex, age, residential environment, and treatment line, this study provides clinically relevant evidence supporting the incorporation of standardized spiritual assessment into routine advanced cancer care.

## 2. Materials and Methods

### 2.1. Purpose

The primary objective of this study was to evaluate the EORTC QLQ-SWB32 as a structured patient-reported spiritual well-being assessment tool in advanced cancer care by performing the formal translation, linguistic validation, and preliminary clinical validation of its Romanian version. Beyond linguistic adaptation, the study aimed to assess the feasibility of integrating spiritual well-being assessment into routine palliative oncology care and to explore the relationship between spiritual well-being and quality of life (QoL) in patients receiving palliative systemic therapy.

### 2.2. Domains

The EORTC QLQ-SWB32 assesses spiritual well-being across four main dimensions: Relationship with Self (RS), Relationship with Others (RO), Relationship with God/the Divine (RSG), and Existential Domain (EX). In addition, the questionnaire includes single-item measures addressing the relationship with God and global spiritual well-being (Table 1).

### 2.3. Scoring Method and Interpretation

The scoring algorithm for the multi-item scales follows the linear transformation principles established for the EORTC QLQ-C30 symptom scales. Raw scores are converted to a standardized scale ranging from 0 to 100, where higher numerical values signify superior levels of spiritual fulfillment and robust functioning within the respective relationship or existential domains.

The instrument includes several non-scoring components: Items 22 and 23 serve as screening criteria for the Relationship with God item, while Items 4, 7, 24, 25, 28, and 29 are utilized for their clinical relevance rather than statistical aggregation. Global Spiritual Well-being was evaluated as a single-item metric using Item 32, which employs a 7-point ordinal response scale anchored by 1 (very poor) and 7 (excellent), with participants choosing fixed whole-number ratings that lacked corresponding descriptive text labels.

### 2.4. Linguistic Validation Procedure

The translation process of the QLQ-SWB32 questionnaire from English into Romanian was conducted in strict accordance with the EORTC Quality of Life Group guidelines. The English source version underwent forward-translation by two independent native Romanian speakers fluent in English. A third investigator performed a reconciliation of these versions, after which the Romanian draft was subjected to back-translation into English by native speakers of both languages. Following EORTC recommendations for linguistic validation, the Romanian version of the EORTC QLQ-SWB32 was pilot-tested with ten cancer patients representing diverse age groups and educational levels. Data collected during this phase were utilized exclusively for the validation process and were omitted from the final study results. All ten participants completed the questionnaire independently; none required further clarification regarding the concepts presented in the text. No patient experienced difficulties in comprehending the lexicon or syntax, and a consensus was reached that the items were easily understood.

### 2.5. Study Design

We conducted a prospective, observational, cross-sectional study in the Medical Oncology Clinic of the Regional Institute of Oncology in Iași, Romania. The study was designed as a single-center clinical validation and feasibility assessment of the Romanian version of the EORTC QLQ-SWB32 in patients with advanced cancer receiving palliative systemic therapy. Data collection was performed over a six-month period, from January 2025 to June 2025, during which 42 patients were enrolled. The study protocol was approved by the institutional Ethics Committee, and all participants provided written informed consent before inclusion.

### 2.6. Study Population

Eligible participants were adults aged 18 years or older with metastatic or locally advanced inoperable cancer who were receiving first-line or subsequent lines of palliative systemic therapy. Patients were excluded if they had cancer treated with curative intent, disease stages other than metastatic or locally advanced inoperable cancer, or clinical conditions that made participation inappropriate, such as severely compromised performance status or acute or chronic complications related to the disease or treatment.

Patients were approached during hospitalization and received a detailed explanation of the study objectives and procedures. Participation was voluntary, and patients were informed that they could withdraw from the study at any time without any impact on their subsequent medical care.

### 2.7. Data Collection Procedure

Questionnaires were administered anonymously and confidentially through structured face-to-face interviews. Participants completed two instruments: the EORTC QLQ-C15-PAL, used to assess quality of life in palliative cancer care, and the EORTC QLQ-SWB32, used to assess spiritual well-being.

Data collection took place during continuous inpatient hospitalization for systemic treatment. Each interview lasted approximately 45 min, allowing sufficient time for completion of both instruments and for clarification of any procedural aspects. Given the personal and potentially sensitive nature of spiritual well-being assessment, participants were offered adequate time to reflect on their responses. They were also given the opportunity to discuss any item or emotional reaction arising during the interview.

A structured case report form was used to collect sociodemographic and clinical data. Sociodemographic variables included age, biological sex, ethnicity, marital status, residential environment, educational attainment, occupational status, and religious affiliation. Clinical variables included primary tumor site, disease stage, and line of systemic therapy for advanced disease, categorized as first-line versus subsequent lines of treatment.

### 2.8. Outcomes

The primary outcomes of the study were the cross-cultural translation, clinical validation, and feasibility assessment of the EORTC QLQ-SWB32 in Romanian-speaking patients with advanced cancer, together with the evaluation of the association between spiritual well-being and QoL in patients receiving palliative systemic therapy.

Secondary outcomes included the exploration of associations between sociodemographic or clinical variables and the four spiritual well-being domains. Particular attention was given to the Relationship with God/the Divine domain, as this dimension may have specific clinical relevance in a culturally religious population and may act as an important correlate of patient-reported well-being.

### 2.9. Statistical Analysis

No formal a priori sample size calculation was performed, as this study was designed as an exploratory investigation with a linguistic validation component.

#### Sample Size Estimation

Although no conventional power calculation anchored to a single confirmatory hypothesis was undertaken, the sample size of each of the two components of this study was nevertheless fixed in advance. The basis for each, and the effect sizes that the resulting sample can detect, are set out below.

For the linguistic validation component, the required sample size was fixed a priori by the EORTC Quality of Life Group translation guidelines followed throughout this study (Section 2.4), which specify that a translated questionnaire should be pilot-tested on a group of 10–15 patients drawn from the target population of the questionnaire and representative in terms of sex, age and education. Ten patients were therefore recruited for the pilot phase, satisfying this requirement; their responses were used exclusively for linguistic validation and were not included in the analyses reported below.

For the clinical validation and feasibility component, the target sample size was defined a priori as the number of consecutive eligible patients who could be recruited at a single center within a predefined six-month accrual window. This procedure yielded 42 patients, 23 receiving first-line and 19 receiving subsequent-line therapy, providing at least 12 patients within each treatment-line stratum. A sample of this size is adequate for a preliminary single-center evaluation of feasibility, acceptability and internal consistency, but it remains well below the several hundred patients required for definitive international field-testing of a questionnaire module under EORTC guidelines, and the study was neither designed nor powered to meet that standard.

To make explicit what this sample size can and cannot detect, a sensitivity (minimum detectable effect) analysis was performed. At a two-sided α of 0.05 and 80% power, the total sample of 42 patients is sufficient to detect a correlation of |*r*| ≥ 0.42. Within treatment-line strata the minimum detectable correlation rises to |*r*| ≥ 0.55 (first line, *n* = 23) and |*r*| ≥ 0.60 (subsequent lines, *n* = 19), and within the smallest sex-, age- and residence-defined subgroups (*n* = 8–12) only very large correlations (|*r*| ≥ 0.72–0.83) are detectable. For two-group comparisons, the detectable standardized mean difference is *d* ≥ 0.89 for the cohort as a whole and *d* ≥ 1.23–1.38 within treatment-line strata. All subgroup analyses are consequently underpowered for small and moderate effects and are reported throughout as exploratory and hypothesis-generating; the absence of statistical significance within these strata should not be read as evidence that no association exists.

To evaluate differences in Global Health Status and RSG scores across various demographic and occupational strata, a series of independent-samples t-tests were performed. This parametric approach was selected due to the continuous nature of the variables and the relative robustness of the t-test to moderate deviations from normality in medium-sized cohorts. Comparisons were conducted for four categorical pairings: Occupational Status (Employed vs. Retired), Residential Environment (Urban vs. Rural), Biological Sex (Male vs. Female) and Age Group (40–64 years vs. >65 years).

Data visualization was achieved using boxplots generated via the ggplot2 and ggpubr packages (R—4.6.1.). To provide simultaneous visual and statistical evidence, *p*-values were computed and overlaid directly onto the plots using the ‘stat_compare_means’ function. To investigate the association between Global Health Status and RSG scores within each specific subgroup, Spearman’s rank correlation coefficients were utilized. This non-parametric method was preferred over Pearson’s correlation to account for potential non-linearity, skewed distributions, and the presence of outliers within smaller subset populations. When spiritual well-being domain scores were compared across subgroups, the Mann–Whitney U test was used, given the exploratory nature of the analysis and the limited subgroup sizes.

Correlation analyses were performed using the cor.test function. Results were visualized through scatterplots differentiated by color for each subgroup. While linear regression lines were superimposed for exploratory and illustrative purposes, no assumptions of strict linearity were made. Correlation coefficients and their corresponding significance levels were annotated directly within the figures to facilitate the comparative interpretation of how demographic factors modulate the link between spiritual well-being and health perception.

All analyses were considered exploratory; therefore, statistically non-significant patterns were interpreted cautiously and discussed only in relation to their potential clinical relevance.

## 3. Results

### 3.1. Patients Characteristics and Clinical Context

The study population comprised 42 patients with advanced cancer receiving palliative systemic therapy at the Regional Institute of Oncology, Iasi, Romania. The cohort had a mean age distribution skewed toward older adulthood, with the majority falling within the 40–64 age group (52.38%), followed by patients aged 65 years and over (40.47%), and a minority aged 18–39 years (7.14%). The sex distribution was approximately balanced, with a slight female predominance (52.38% vs. 47.61%). Most patients resided in urban areas (54.76%) and were retired (54.76%), reflecting the predominantly older demographic of the cohort. Regarding marital status, the majority were married (64.28%), while widowed, divorced, and single patients accounted for 14.28%, 11.90%, and 9.52% of the sample, respectively. In terms of educational attainment, upper secondary education was the most frequently reported level (64.28%), followed by university education (16.66%) and tertiary non-university training (9.52%) (Table 2).

The cohort was highly homogeneous from an ethnic and religious perspective. All participants identified as Romanian and Christian, with most patients belonging to the Eastern Orthodox denomination (92.85%). Smaller proportions identified as Roman Catholic (4.76%) or Baptist (2.38%) (Table 3). This socio-cultural profile is relevant for the interpretation of spiritual well-being, particularly for domains related to the relationship with God/the Divine.

From a clinical perspective, 54.76% of patients were receiving first-line palliative systemic therapy, whereas 45.23% were receiving subsequent lines of treatment. Gastrointestinal malignancies represented the most frequent cancer group, followed by gynecological, genitourinary, and lung cancers. Less frequent diagnoses included sarcoma, breast cancer, and head and neck cancer. Regarding performance status, the majority of patients (59.52%) presented with an ECOG PS of 1, followed by those with an ECOG PS of 0 (16.66%) and an ECOG PS of 2 (23.80%). The inclusion of patients across different treatment lines allowed an exploratory evaluation of spiritual well-being in relation to disease trajectory and cumulative treatment exposure (Table 4).

### 3.2. Feasibility and Acceptability of Spiritual Well-Being Assessment

The Romanian version of the EORTC QLQ-SWB32 was pre-tested in ten patients with cancer, representing different demographic and educational backgrounds. Data obtained during this pilot phase were used exclusively for linguistic validation and were not included in the final study analysis.

Overall, the questionnaire demonstrated good feasibility and acceptability. All participants were able to complete the instrument independently, and no item was identified as linguistically unclear or conceptually difficult to understand. Patients generally considered the inclusion of spiritual well-being assessment relevant within holistic cancer care.

One participant noted that Item 30, “I believe in life after death,” could potentially evoke existential distress by reminding patients of their proximity to the end of life. However, no adverse emotional reactions were observed during administration. These findings support the clinical acceptability of the instrument, while also emphasizing the importance of administering spiritual well-being questionnaires in a sensitive and supportive context.

### 3.3. Exploratory Associations Between Spiritual Well-Being and Quality of Life According to Treatment Line

Exploratory analyses were conducted after stratifying patients according to treatment line for advanced disease: first-line palliative systemic therapy versus subsequent lines of treatment. This stratification was selected to explore whether the association between quality of life and spiritual well-being differs across the disease trajectory, particularly in relation to cumulative treatment burden, disease progression, and repeated exposure to adverse clinical events.

Particular attention was given to the Relationship with God/the Divine domain, as this dimension was considered especially relevant in the socio-cultural context of the study cohort. Across subgroup analyses, the association between Global Health Status and RSG scores showed several clinically meaningful patterns, even when between-group differences did not reach statistical significance.

### 3.4. Sex-Based Patterns in the Relationship Between RSG and Quality of Life

Independent-samples *t*-tests did not identify statistically significant differences in Global Health Status or RSG scores according to biological sex. However, correlation analyses suggested a positive association between RSG and Global Health Status in both male and female patients.

**Figure 1:**
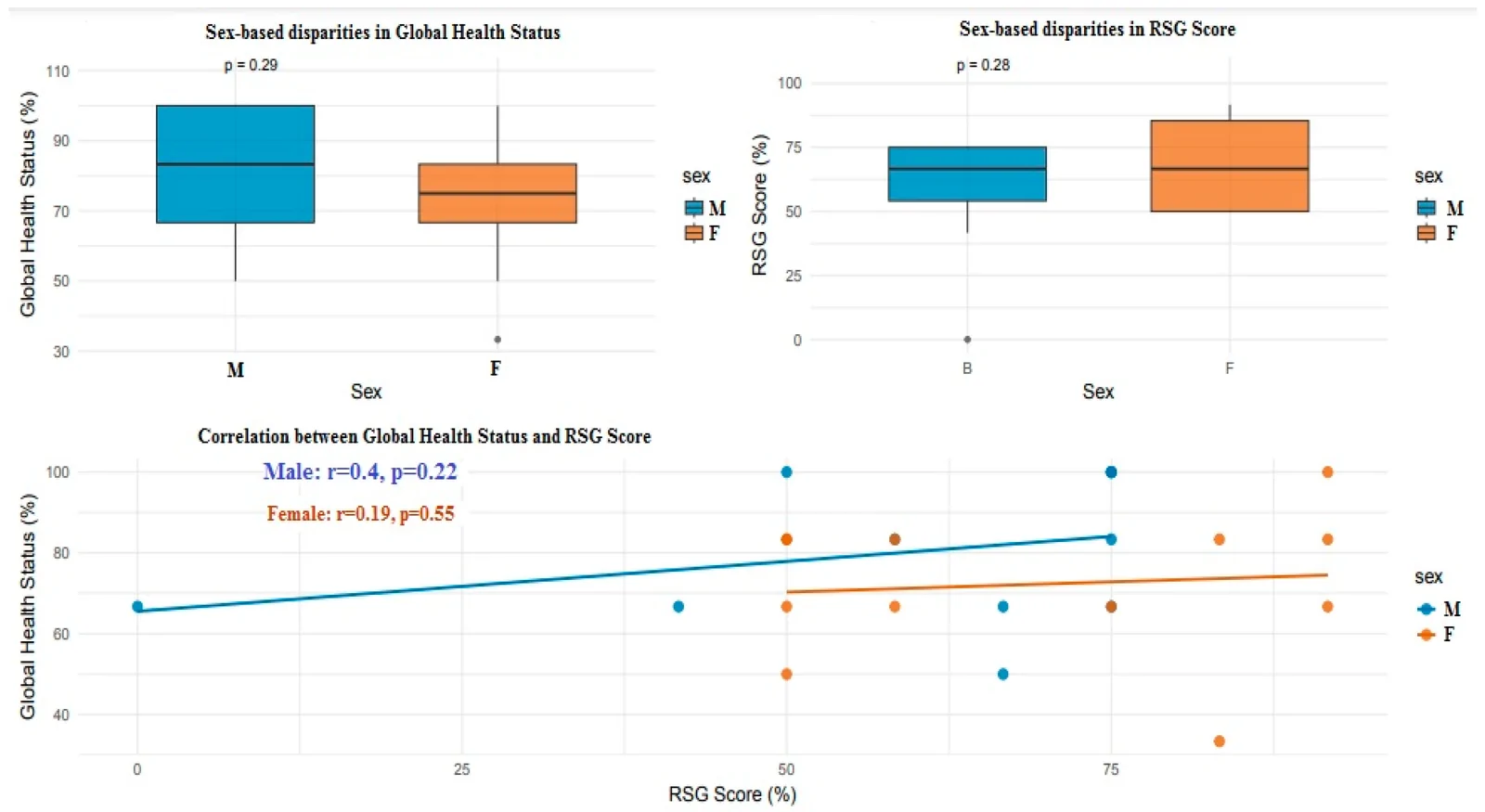
Correlation between Global Health Status and RSG Score in First-Line Treatment, Stratified by Sex. (Male: r = 0.4 (positive correlation); *p* = 0.22 (not statistically significant); Female: r = 0.19 (positive correlation); *p* = 0.55 (not statistically significant).

Among patients receiving first-line therapy, male patients showed a stronger correlation between RSG and Global Health Status than female patients, suggesting that the relationship with God/the Divine may be more closely associated with perceived quality of life in men at this stage of the treatment trajectory (Figure 1).

**Figure 2:**
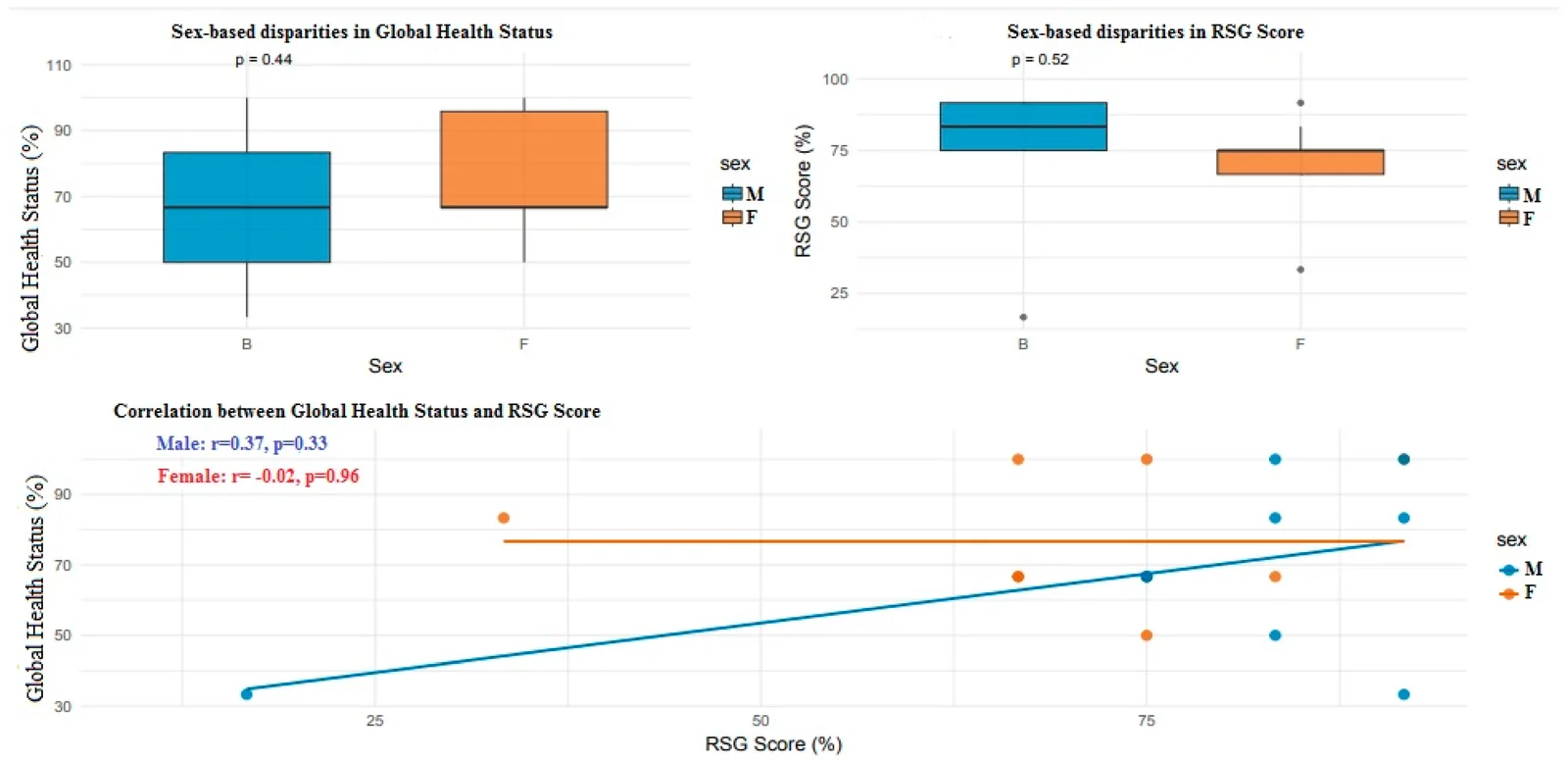
Correlation between Global Health Status and RSG Score in Subsequent Lines of Treatment, Stratified by Sex. (Male: r = 0.37 (positive correlation); *p* = 0.33 (not statistically significant); Female: r = −0.02 (negative correlation); *p* = 0.96 (not statistically significant)).

This pattern became more evident among patients receiving subsequent lines of therapy, where the association between RSG and Global Health Status appeared stronger in male patients than in female patients (Figure 2). Although exploratory, these findings suggest that the relationship with God/the Divine may become increasingly relevant to perceived quality of life in male patients as the disease trajectory becomes more prolonged or treatment-exposed.

### 3.5. Age-Based Patterns in the Relationship Between RSG and Quality of Life

Independent-samples t-tests did not show statistically significant differences in Global Health Status or RSG scores between patients aged 40–64 years and those aged over 65 years. Nevertheless, correlation analyses revealed distinct age-related patterns according to treatment line.

**Figure 3:**
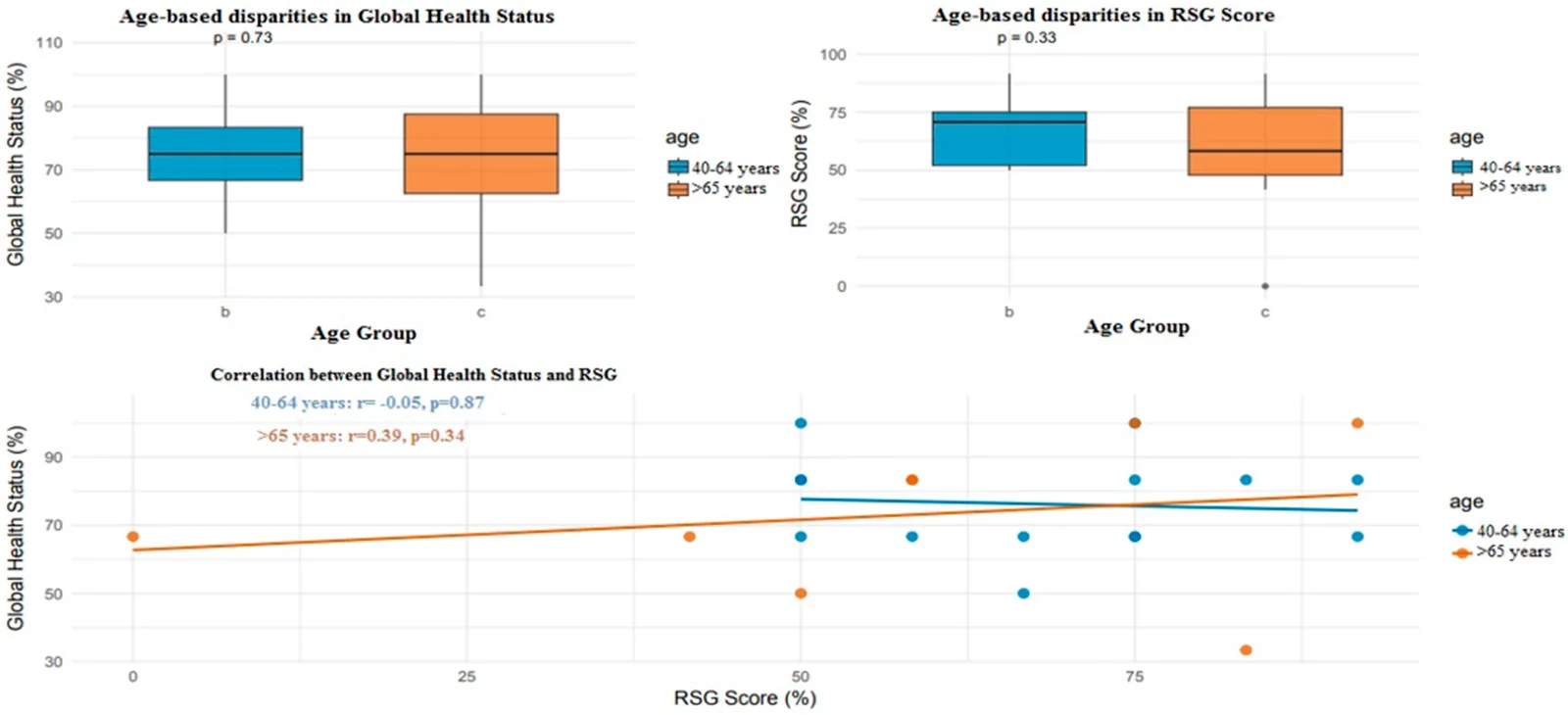
Correlation between Global Health Status and RSG Score in First Line Treatment, Stratified by Age Group. (40–64 years: r = 0.37 (positive correlation); *p* = 0.37 (not statistically significant); >65 years: r = −0.12 (negative correlation); *p* = 0.76 (not statistically significant)).

Among patients receiving first-line palliative systemic therapy, a positive association between RSG and Global Health Status was observed in patients aged over 65 years. In contrast, patients aged 40–64 years showed a subtle negative correlation in the same treatment setting (Figure 3).

**Figure 4:**
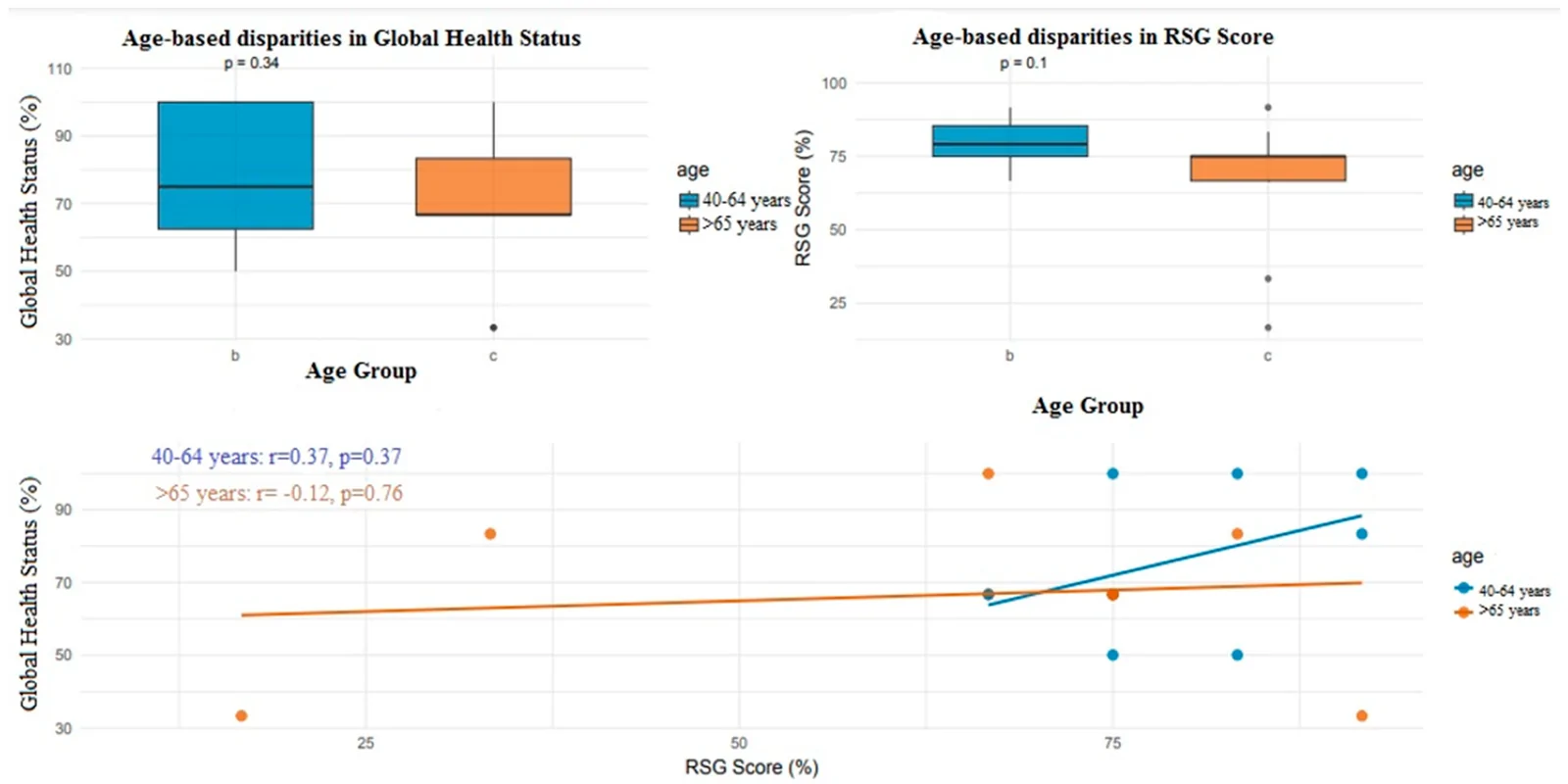
Correlation between Global Health Status and RSG Score in Subsequent Lines of Treatment, Stratified by Age Group. (40–64 years: r = −0.05 (negative correlation); *p* = 0.87 (not statistically significant); >65 years: r = 0.39 (positive correlation); *p* = 0.34 (not statistically.

An opposite pattern was observed among patients receiving subsequent lines of therapy. In this subgroup, patients aged 40–64 years demonstrated a positive association between RSG and Global Health Status, whereas patients aged over 65 years showed a weak negative correlation (Figure 4). These exploratory findings suggest that the relationship between spiritual well-being and perceived quality of life may evolve differently across age groups as patients progress through the palliative treatment trajectory.

### 3.6. Residential Environment and RSG-Quality-of-Life Associations

In patients receiving first-line palliative systemic therapy, no statistically significant difference in Global Health Status or RSG scores was observed between urban and rural residents. However, a positive association between RSG and Global Health Status was identified in both residential subgroups. In this setting, the correlation appeared more pronounced among urban residents compared with rural residents.

Among patients receiving subsequent lines of systemic therapy, the association between QoL and residential environment reached statistical significance. In contrast to the first-line subgroup, rural residents showed a stronger association between RSG and Global Health Status. This pattern may indicate that the clinical relevance of spiritual well-being differs according to residential environment and may become more pronounced in rural patients as treatment lines advance.

### 3.7. Domain-Level Patterns of Spiritual Well-Being

The four spiritual well-being domains were further explored according to sex using the Mann-Whitney U test. Most comparisons did not reach statistical significance; however, descriptive patterns were consistent with the correlation analyses. Male patients showed higher mean scores across three of the four domains, whereas female patients had marginally higher scores in the existential domain.

The observed difference in the RSG domain is clinically relevant in the context of the positive association between this domain and Global Health Status. In contrast, scores for the Relationship with Self domain differed only slightly between male and female patients.

Age-related domain patterns were also observed. Patients aged over 65 years showed a trend toward higher scores in the Relationship with Self domain compared with patients aged 40–64 years. This may suggest that older patients are more oriented toward introspection, self-acceptance, or inner reconciliation during advanced cancer care. These domain-level findings should be interpreted cautiously, given the exploratory design and limited sample size, but they provide useful insight into how different dimensions of spiritual well-being may vary across patient subgroups.

## 4. Discussion

This study evaluated spiritual well-being as a structured patient-reported assessment dimension in patients with advanced cancer receiving palliative systemic therapy. The association between the Relationship with God/the Divine domain and QoL varied across sociodemographic and clinical subgroups, suggesting that spiritual well-being may have different clinical relevance depending on patient characteristics and treatment trajectory. Stronger RSG-QoL correlations were observed among male patients, patients aged 40–64 years receiving subsequent lines of therapy, and rural patients in later treatment lines. These findings should be interpreted as exploratory, but they support the potential value of incorporating spiritual well-being assessment into individualized palliative oncology care.

The National Comprehensive Cancer Network (NCCN) guidelines suggest that the assessment of spiritual distress plays a pivotal role in the screening for psychosocial disorders among cancer patients. Nevertheless, this dimension remains among the least explored due to a lack of definitive conceptualization and the inherent challenges of quantifying subjective data. Furthermore, there is a consensus that addressing religiosity and spirituality requires a multidimensional evaluation, including the beliefs and practices of the patient’s religious community, individual experiences, the search for meaning, and the connection with self, others, and the sacred, as outlined in the framework provided by the National Consensus Project (NCP) for Quality Palliative Care [25].

Spiritual struggle or religious conflict is a frequently encountered entity that encompasses intrapersonal, interpersonal, and divine domains [26]. These conflicts may originate from both the individual’s psychological mindset and core belief systems. For instance, specific patient cohorts may experience feelings of fear or guilt regarding their suffering or may perceive a sense of abandonment by a non-responsive sacred entity [27].

The alleviation of spiritual suffering has been associated with enhanced QoL, preservation of psychological and social functionality, and reductions in depression and anxiety among patients with cancer. Conversely, unmet spiritual needs have been linked to lower hospice care utilization and increased healthcare system costs in terminally ill patients [25].

Patients facing incurable malignancies often demonstrate a significant orientation toward spiritual and religious dimensions, sometimes perceiving “peace with God” as clinically comparable to effective symptom management. Rabow et al. conducted a prospective study evaluating spiritual well-being among cancer patients receiving systemic treatment and supportive care across diverse religious denominations. Their results indicate that improvements in spiritual well-being are not significantly associated with formal affiliation to a religious community; rather, they are optimized when physicians or nursing staff consistently dedicate time to discussing spiritual themes, even without the involvement of a chaplain or clergy member. Objectively, this improvement in spiritual well-being was reflected by measurable reductions in anxiety, depression, and cancer-related fatigue [28].

In an observational study validating the EORTC QLQ-SWB32 within a Croatian population, significant differences were identified across predefined sociodemographic groups. The instrument was administered to patients with early-stage malignancies at various phases of the oncological trajectory. Their results demonstrated higher scores in the existential and RSG domains among women compared with men, as well as in participants under 50 years of age compared with the elderly cohort. In contrast, our findings suggest a divergent pattern regarding the association between sex and spiritual well-being. Specifically, our data indicate that men had higher RSG levels irrespective of treatment line, and that the correlation between this dimension and global health status was more robust. Regarding age, patients aged 40–64 years undergoing subsequent lines of therapy exhibited both higher RSG scores and a stronger correlation between spirituality and QoL. Conversely, in the first-line treatment subgroup, these findings were reversed, favoring the cohort aged over 65 years [29].

In this cohort, the 40–64 age group exhibited a positive correlation between QoL and RSG, whereas the older group demonstrated a negative correlation. Given the socio-cultural traditions of the Romanian context and the predominantly Orthodox Christian affiliation of the participants, these findings may suggest that middle-aged adults tend to rely more strongly on religiosity when confronted with prolonged and profound suffering. Religious psychological coping may increase QoL, as indicated by Tarakeshwar et al., and may reduce fear of death, which is known to be elevated in adults aged 40–64 years [30,31]. Two other variables that may influence the outcomes of the study might be cancer stigma and cancer literacy. Ayşïn et al. described that cancer stigma negatively affects women’s psychological well-being and intensify the turn toward spiritual meaning-making and the use of spirituality as a coping mechanism [32]. Religiosity can reduce the impact of stigma on mental well-being of cancer patients [33]. Cancer literacy impacts the quality of life in patients with cancer. Those patients who have inadequate cancer literacy may find difficult to understand medical information and manage their treatment, resulting in exacerbated treatment-related symptoms [34]. Information about the health condition and religious beliefs were found to be important coping mechanisms in dealing with the suffering caused by cancer [35]. Gabriel et al. indicated that patients with cancer need information and spiritual support to help them manage this health condition [36].

Among men, the relationship with a benevolent and kindly God may be particularly relevant, as illness may be interpreted in relation to God’s will and integrated into broader aspects of life. When religion is used as a coping strategy by men, it may become a prominent mechanism for managing difficult emotions [37].

A systematic review published in 2018 aimed to identify and assess the association between relational spirituality and QoL. The final analysis synthesized findings from 20 studies conducted over a decade (2007–2017), encompassing a total population of 132,053 patients. Most subjects were diagnosed with incurable chronic conditions, including various malignancies. The review identified several patterns of association between these two constructs; although neutral, negative, or non-existent associations were also reported, the most frequent finding was a significant positive correlation. Notably, the study populations were not limited to oncology but also included patients with other incurable conditions, such as dementia, schizophrenia, stage V chronic kidney disease requiring hemodialysis, and HIV/AIDS. Furthermore, the review noted that data regarding religious affiliation were highly heterogeneous, precluding a definitive classification by denomination [38].

Another review, which synthesized 53 articles, including 12 randomized controlled trials involving 13,590 cancer patients, sought to elucidate the mechanisms through which spirituality may enhance QoL in this population. The findings support the integration of spirituality as a fundamental pillar of holistic care for all patients, irrespective of specific religious practices or communal affiliations. While the impact of religion and spirituality is predominantly positive, the review also highlights that these constructs can, in certain contexts, negatively affect QoL through spiritual distress, manifested as feelings of abandonment or anger directed toward the Divine. Overall, this publication reaffirms the important influence of spiritual well-being on global health status and QoL in patients navigating the complexities of a cancer diagnosis [39].

Consistent with findings reported in international cohorts, the results derived from our population suggest a positive association between the Relationship with the Divine domain of spiritual well-being and overall QoL across most sociodemographic subgroups.

A systematic review of 26 randomized controlled trials (N = 2580) assessed spiritual interventions in cancer patients, with 50% of studies focusing on advanced-stage disease. Various individual and group modalities, including Meaning-Centered Psychotherapy, meditation, and Dignity Therapy, were found to significantly enhance spiritual well-being, life meaning, and overall QoL [40]. While Izgu’s meta-analysis indicates that these interventions do not significantly mitigate physical symptom burden, earlier evidence suggests that they may reduce depression and anxiety, indirectly optimizing both spiritual well-being and global QoL [41].

Recent empirical evidence underscores the significant role of spirituality in clinical outcomes. A systematic review of 36 studies established a strong, independent correlation between spiritual well-being and QoL. This association is primarily driven by two factors: meaning and peace. Furthermore, improvements in QoL were linked to patients’ existential perspectives and mental health status [42]. In a clinical context, research involving 120 African American cancer patients demonstrated that spiritual well-being functions as a protective factor against cancer pain. By mitigating the perception of pain and associated symptoms, spiritual health may directly enhance overall QoL in palliative settings [18].

A multicentric study conducted across the United States of America and South America evaluated 325 Latino-American patients with advanced cancer in palliative and oncological settings. The cohort exhibited a profound spiritual and religious identity, with 97% identifying as spiritual and 89% as active religious practitioners, primarily within the Christian faith (>95%). Following a cancer diagnosis, 44% of subjects reported an intensification of their spiritual life, while only 8% reported a decline. This suggests that existential distress often triggers a shift toward spiritual and religious coping mechanisms. The increase in spirituality after diagnosis was characterized by a strengthened connection with the divine, interpersonal relationships, family or community, and the inner self. A significant majority of participants perceived their spiritual or religious convictions as contributing to their overall survival [12].

In our study, the correlation between QoL and spiritual connectivity appeared stronger among male patients, patients aged 40–64 years, and rural patients receiving subsequent treatment lines. These exploratory findings suggest that prolonged disease duration and therapeutic exhaustion may intensify the reliance on transcendental frameworks, where the relationship with God/the Divine becomes increasingly relevant for coping, meaning-making, and perceived well-being.

A study conducted by Astrow et al. aimed to identify the spiritual and psychosocial requirements of cancer patients, revealing that a significant majority (79%) reported at least one spiritual need, while 80% identified concurrent psychosocial needs. The findings indicated a higher prevalence of existential needs compared with formal religious ones, which were reported by less than half of the participants. A critical finding of the study was that 25% of patients reported unmet spiritual needs, with 11% requesting support to address these deficits. This suggests that while spiritual distress is prevalent, a significant number of patients remain underserved by current cancer care models. These data highlight a clear distinction between institutional religion and personal spirituality. For most cancer patients, the priority lies in finding hope and meaning rather than formal clerical intervention. This underscores the necessity for healthcare providers to integrate spiritual screening into routine clinical practice to address the proportion of patients facing existential distress without adequate support [43].

Taken together, these findings suggest that spiritual well-being is not only a subjective experience but also a clinically relevant patient-reported dimension. By contributing to patient-reported outcomes such as anxiety, hope, sleep, and perceived QoL, spiritual care may represent a meaningful component of comprehensive palliative and cancer care [40].

## 5. Study Strengths and Limitations

Several key strengths and limitations arise from our research. The linguistic validation of the EORTC QLQ-SWB32 was carried out in Romanian, thereby establishing a culturally and semantically appropriate version for Romanian-speaking populations. As a result, this instrument may be used not only in Romania, but also in the Republic of Moldova and within Romanian-speaking communities across Europe and the United States.

The questionnaire was administered in balanced proportions across the predefined subgroups, ensuring equitable representation according to sex, age category, place of residence, and treatment setting. This structured distribution aimed to enhance the comparability of responses and to support the interpretability of subgroup analyses.

While these methodological strengths support the applicability of the instrument, several limitations should be acknowledged. In particular, the relatively small sample size (n = 42) constrains the statistical power of the analysis and may limit the generalizability of the findings. Consequently, further validation in a larger cohort would be necessary to consolidate the psychometric robustness of the instrument and the clinical applicability of the observed associations.

In addition, the cross-sectional design entails that participants were assessed at a single time point, precluding the evaluation of temporal stability and responsiveness to change. To enhance the longitudinal validity and reliability of the instrument, future research should incorporate follow-up assessments at predefined intervals, thereby allowing for a more comprehensive evaluation of its performance over time.

From a clinical perspective, the systematic assessment of spiritual well-being may help identify patients with advanced cancer who experience hidden vulnerability despite apparently stable clinical status. The Relationship with the Divine domain may be particularly relevant in culturally religious populations, where spiritual coping can influence perceived quality of life, emotional adaptation, and the need for supportive care. Incorporating a standardized spiritual well-being instrument into routine palliative oncology assessment could therefore support earlier recognition of spiritual distress and guide referral toward psycho-oncology, palliative care, or spiritual care services.

## 6. Future Directions

To build on these exploratory results, future research should pursue an adequately powered, prospective multicenter study. A stratified longitudinal design, incorporating pre-planned controls for multiple testing, will allow us to formally confirm the scale’s four-factor structure, assess its broader psychometric properties against international standards, and establish its test–retest reliability and responsiveness over time. This design will also enable rigorous testing of key hypotheses, including the link between subjective well-being and quality of life. Finally, expanding recruitment to include religiously diverse and secular cohorts will ensure the instrument is valid beyond predominantly Orthodox populations before advancing to international field-testing.

## 7. Conclusions

This study supports the clinical validation and feasibility of the Romanian version of the EORTC QLQ-SWB32 as a structured patient-reported spiritual well-being assessment tool in patients with advanced cancer. The instrument demonstrated linguistic clarity, conceptual equivalence, and clinical acceptability, supporting its potential use in routine palliative oncology practice.

Spiritual well-being, particularly the Relationship with God/the Divine domain, was clinically associated with overall quality of life, with variations according to sex, age, residential environment, and treatment trajectory. These findings suggest that spiritual needs may evolve along the illness continuum and that standardized spiritual assessment could help identify patients requiring tailored supportive care.

Future studies involving larger and more diverse cohorts are needed to confirm these exploratory findings and to further define the role of spiritual well-being assessment in advanced cancer care.

## Figures and Tables

**Table 1:** Correlations between EORTC QLQ-SWB32 domains and individual items.

Assessment Scale	Number of Items
RO (8, 9, 10, 11, 12, 13)	6
RS (5, 6, 17, 18, 19)	5
RSG (20, 21, 27, 30, 31)	5
EX (1, 2, 3, 14, 15, 16)	6
Relationship with God (26)	1
Global SWB (32)	1

RO—Relationship with Others; RS—Relationship with Self; RSG—Relationship with God/the Divine; EX—Existential Domain; SWB—Spiritual Well-being.

**Table 2:** Sociodemographic Characteristics (N = 42).

	Total (N = 42)	%
**Age (years)**		
18–39	3	7.14
40–64	22	52.38
>65	17	40.47
**Sex**		
Female	22	52.38
Male	20	47.61
**Residence**		
Rural	19	45.23
Urban	23	54.76
**Marital Status**		
Single/Never Married	4	9.52
Married	27	64.28
Divorced	5	11.90
Widowed	6	14.28
**Education**		
Primary Education	2	4.76
Middle School	3	7.14
Upper Secondary	27	64.28
Tertiary Non-University	4	9.52
University	7	16.66
**Occupational Status**		
Employed/Freelancer	15	35.71
Retired	23	54.76
Household	3	7.14
Unemployed	1	2.35

**Table 3:** Socio-cultural Characteristics (N = 42).

	Total (N = 42)	%
**Ethnicity**		
Romanian	42	100
Other	0	0
**Religion**		
Christianity	42	100
Other	0	0
**Religious Denomination**		
Orthodox	39	92.85
Roman Catholic	2	4.76
Baptist	1	2.38

**Table 4:** Clinical Characteristics (N = 42).

	Total (N = 42)	%
Line of Palliative Systemic Therapy		
First Line Therapy	23	54.76
Subsequent Lines	19	45.23
Cancer Type		
Gastrointestinal Cancers	21	47.61
Pancreatic Cancer	7	16.66
Colorectal Cancer	9	21.42
Esophageal/Gastric Cancers	5	11.90
Gynecological Cancers	6	14.28
Endometrial Cancer	5	11.90
Cervical Cancer	1	2.38
Genitourinary Cancers	5	11.90
Prostate Cancer	2	4.76
Renal Cancer	1	2.38
Testicular Cancer	2	4.76
Lung Cancer	5	11.90
Sarcoma	3	7.14
Breast Cancer	1	2.38
Head and Neck Cancer	1	2.38
ECOG PS		
0	10	23.80
1	25	59.52
2	7	16.66

## Data Availability

Data available on request due to restrictions. We are unable to provide or publicly deposit the individual-level minimal dataset, including for external transfer or editorial evaluation. The dataset contains sensitive clinical, sociodemographic, quality-of-life, and spiritual well-being information collected from patients with advanced cancer. Given the relatively small sample size and the combination of demographic and clinical variables, removal of direct identifiers would not completely eliminate the risk of participant re-identification. Moreover, the informed consent obtained from participants covered their participation in the research and the publication of study results, but it did not include consent for the transfer, public deposition, or sharing of individual-level data with third parties. The ethical approval also requires the confidentiality of the patient information accessed and analysed during the project. Consequently, transferring the individual-level dataset would extend beyond the conditions under which the data were collected and approved. All aggregated data required to support the main findings are presented in the manuscript, tables and figures.
